# Ultrafast laser surgery probe for sub-surface ablation to enable biomaterial injection in vocal folds

**DOI:** 10.1038/s41598-022-24446-5

**Published:** 2022-11-29

**Authors:** Liam Andrus, Hamin Jeon, Michal Pawlowski, Benoit Debord, Frederic Gerome, Fetah Benabid, Ted Mau, Tomasz Tkaczyk, Adela Ben-Yakar

**Affiliations:** 1 Department of Biomedical Engineering, The University of Texas at Austin, Austin, TX 78712 USA; 2 Department of Bioengineering, Rice University, Houston, TX 77005 USA; 3 GPPMM Group, XLIM, CNRS-University of Limoges, Limoges, France; 4 Department of Otolaryngology-Head and Neck Surgery, The University of Texas Southwestern Medical Center, Dallas, TX 75390 USA; 5 Department of Mechanical Engineering, The University of Texas at Austin, Austin, TX 78712 USA

**Keywords:** Surgery, Biomedical engineering, Ultrafast lasers, Nonlinear optics, Optomechanics, Preclinical research

## Abstract

Creation of sub-epithelial voids within scarred vocal folds via ultrafast laser ablation may help in localization of injectable therapeutic biomaterials towards an improved treatment for vocal fold scarring. Several ultrafast laser surgery probes have been developed for precise ablation of surface tissues; however, these probes lack the tight beam focusing required for sub-surface ablation in highly scattering tissues such as vocal folds. Here, we present a miniaturized ultrafast laser surgery probe designed to perform sub-epithelial ablation in vocal folds. The requirement of high numerical aperture for sub-surface ablation, in addition to the small form factor and side-firing architecture required for clinical use, made for a challenging optical design. An Inhibited Coupling guiding Kagome hollow core photonic crystal fiber delivered micro-Joule level ultrashort pulses from a high repetition rate fiber laser towards a custom-built miniaturized objective, producing a 1/e^2^ focal beam radius of 1.12 ± 0.10 μm and covering a 46 × 46 μm^2^ scan area. The probe could deliver up to 3.8 μJ pulses to the tissue surface at 40% transmission efficiency through the entire system, providing significantly higher fluences at the focal plane than were required for sub-epithelial ablation. To assess surgical performance, we performed ablation studies on freshly excised porcine hemi-larynges and found that large area sub-epithelial voids could be created within vocal folds by mechanically translating the probe tip across the tissue surface using external stages. Finally, injection of a model biomaterial into a 1 × 2 mm^2^ void created 114 ± 30 μm beneath the vocal fold epithelium surface indicated improved localization when compared to direct injection into the tissue without a void, suggesting that our probe may be useful for pre-clinical evaluation of injectable therapeutic biomaterials for vocal fold scarring therapy. With future developments, the surgical system presented here may enable treatment of vocal fold scarring in a clinical setting.

## Introduction

Vocal fold (VF) scarring is a primary cause of voice disorders^1,2^. As an undesirable consequence of surgical excision of VF lesions, VF scarring can result in severe dysphonia and negatively impact quality of life^3,4^. There is currently no effective treatment for chronically scarred VF^5^. The lamina propria, a sub-epithelial tissue layer consisting primarily of collagen, elastin, and reticulin fibers, is largely responsible for the VF vibratory phenomenon and is highly sensitive to scar formation. Many hydrogel-based biomaterials have been developed to repair scarred VF^6–9^, however sub-optimal localization results in poor treatment repeatability^10–14^. Problems arise during injection into the superficial lamina propria (SLP), as the injected biomaterial tends to infiltrate around rather than into the stiff scar tissue. Thus, there is a need for a method to precisely localize biomaterials within the scarred SLP while avoiding any additional scar formation.

The ultrafast laser ablation process relies on rapid multiphoton absorption at the focal plane, resulting in sub-focal volume energy confinement and minimal thermal damage to surrounding tissues^15–18^. Such a high degree of spatial and thermal confinement enables precise material removal inside bulk tissues. To address the challenges of VF scarring, our group proposed a treatment in which a biomaterial injection space is created within the SLP via ultrafast laser ablation^19–21^. Using a benchtop microscope equipped with a 0.75 numerical aperture (NA) objective and a high repetition rate femtosecond fiber laser, Hoy et al*.* demonstrated sub-epithelial void formation in excised porcine VF ~ 100 μm beneath the epithelial surface^19^ which is well within the SLP, considering the epithelial thickness of human, canine, and porcine VF is typically 50–80 μm^22,23^. Further ex vivo studies by Hoy et al*.* showed injection of a model biomaterial into ablated voids created in excised scarred hamster cheek pouches^20^. The authors showed that injection of a model biomaterial (PEG30) into voids significantly reduced back-flow and improved localization when compared to biomaterial injection into scar tissue only. More recently, Gabay et al*.* demonstrated long-term retention of PEG30 within sub-epithelial voids created in an in vivo scarred hamster cheek pouch model^24^. Using the same fiber laser and benchtop system as Hoy et al*.*, Gabay and coauthors found that PEG30 remained within the voids for a period of up to two weeks, suggesting that injection into voids improved long-term biomaterial retention. While these results were encouraging, the large optics (i.e., microscope objective, galvo scanning mirror pair, scan/tube lenses etc.) and free space delivery of laser light limited clinical translation. Thus, flexible delivery of tightly focused ultrashort pulses through miniaturized optical systems is required to translate our VF scarring therapy to the clinic.

Towards clinically viable ultrafast laser surgery, various miniaturized surgical probes have been developed^25–30^. These probes used low NA focusing optics to enable rapid surface ablation of soft and hard tissues. During ultrafast laser ablation, a weakly focused beam necessitates high peak powers to exceed tissue ablation thresholds. Due to the presence of tissue scatterers, even higher peak powers are required when targeting sub-surface structures. Beyond a tissue-specific critical peak-power, self-focusing can cause the beam to collapse above the targeted focal plane, leading to ablation of superficial tissue layers^31^. Thus, high NA focusing of ultrashort laser pulses is required to initiate void formation across a sub-epithelial plane while avoiding the deleterious effects of self-focusing. Additionally, as scarring on the medial VF surface is more clinically relevant for voice production, the forward-firing architecture of previous probe iterations is ill-suited for the proposed scarred VF therapy.

In this paper, we present an ultrafast laser surgery probe designed to translate our scarred VF therapy to the clinic. The probe described here improves upon past designs by employing a miniaturized high NA objective to facilitate sub-epithelial tissue ablation. We utilize a side-firing architecture and an external actuation system to enable automated scanning across a large sub-epithelial plane in a form factor suitable for endoscopic laryngeal surgery. To demonstrate the probe’s surgical performance, we perform sub-epithelial void formation and biomaterial injection in excised porcine hemilarynges towards a viable therapy for scarred human VF.

## Experimental approach

**Figure 1:**
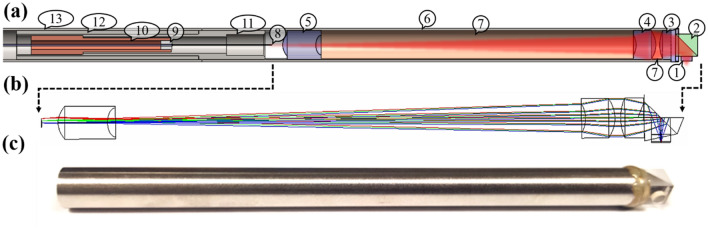
Overview of ultrafast laser surgery system. (**a**) Opto-mechanical design of surgery probe. The probe focuses the laser beam (red trace) onto a sub-surface tissue plane using a miniaturized objective consisting of a sapphire window (1), reflective microprism (2), ZnS lens (3), and two CaF_2_ lenses (4 and 5). All optical components are housed within a 304SS hypodermic tube (6) and distances between the three lenses are set by brass spacers (7). The locus of the scanned Kagome fiber tip (8) is mapped in Zemax to simulate the path of the rays from the focal plane back through the objective. A PMMA insert (9) centered the fiber within the inner cavity of a piezo-electric tube (10). The piezo electric tube (10) was centered within an 304SS inner casing (11) using an accurately turned epoxy plug. The inner casing (11) was centered within the hypodermic tube (6) to align the piezo-HCPCF assembly to the miniaturized objective. Two outer casings (12, 13) were added to secure the backend of the probe to the miniaturized objective. (**b**) Optical schematic of miniaturized objective. A ray trace depicts different launch angles from the fiber tip through the system. (**c**) Final assembly and packaging of miniaturized objective in hypodermic tube.

The surgical probe must meet multiple key requirements for clinical adoption: 1) Flexible delivery of ultrashort laser pulses to the surgical region of interest (ROI), 2) high NA focusing optics to avoid out of focus damage, 3) side-firing architecture, 4) large-area beam scanning to enable formation of large sub-epithelial voids, and 5) a small form factor for integration with existing microlaryngeal surgical instrumentation. Considering all these aspects, we designed a miniaturized probe as illustrated in Fig. 1a.

### Kagome HCPCF and piezo-fiber scanning assembly

To provide flexible delivery of laser light to the surgical probe, we used a Kagome-type hollow core photonic crystal fiber (HCPCF) developed by Fetah Benabid’s research group. Kagome HCPCFs are ideal waveguide candidates for ultrafast laser surgery due to their high-peak power handling capabilities, low group velocity dispersion (GVD), and efficient transmission of ultrashort NIR pulses^32^. Specifically, we used a 7 cell, ~ 52 μm inner core diameter, 6 m long Kagome HCPCF which exhibits low GVD (–437 fs^2^/m) and low attenuation (0.1 dB/m) at 1035 nm. The distal HCPCF length was integrated into a piezo-based Lissajous beam steering element, similar to previous studies^27,29,30^. To build the piezo-HCPCF assembly, we initially soldered leads onto the proximal end of a four-quadrant piezo-ceramic tube (EBL Photonics Inc.), coated the leads with epoxy, and diamond-turned the epoxy plug to match the inner diameter of the corresponding metal housing. We then centered a 17 mm length of the Kagome HCPCF tip out of the distal end of the piezo-ceramic tube using a precision machined PMMA insert. After alignment, we glued the HCPCF, PMMA insert, and piezo tube together using epoxy. We encased the proximal end of the fiber within plastic tubing (FT020, Thorlabs) to prevent fiber breakage prior to integrating the piezo-fiber assembly into the external actuation assembly (Sect. “Mechanical design”) for further testing.

### Miniaturized objective design

The surgical probe requires high NA focusing of ultrashort laser pulses delivered through the low NA Kagome HCPCF to initiate localized ablation below the VF epithelium, necessitating the development of custom miniaturized focusing optics. Additionally, the high pulse energies needed for ablation and the small form factors required for clinical use require careful selection of lens materials to avoid optical nonlinearities within the system. We used Zemax for design, optimization, and tolerance analysis of a miniaturized objective^33^. The probe’s side-firing architecture required that the geometric distance between the focal plane and the nearest lens surface was ≥ 4 mm. This constraint, in addition to the high NA and small form factor required for clinical use, made for a challenging optical design. The objective was optimized for the 1035 nm central wavelength of our laser light source, ≤ 6 mm lens diameters to ensure a small form factor, and seawater immersion to provide focusing in tissue. To avoid self-focusing effects during surgery, we optimized the objective design for the highest possible tissue-side NA while also considering lens fabrication tolerances to ensure manufacturability. Finally, we targeted a working distance of > 100 μm to enable localized void formation below the VF epithelium.

The final objective design (Table 1) had a nominal tissue-side NA of 0.47 and a working distance of 112 μm when focusing in seawater, meeting our design specifications. To estimate the theoretical focal plane beam size, we used the diffraction encircled energy (DEE) module in Zemax. Here, input beam parameters were defined by the Kagome HCPCF, considering Gaussian beam truncation at the optical system’s minimum clear aperture. We estimated a 1*/e*^*2*^ focal spot radius of 0.94 μm across a 47 × 47 µm^2^ FOV for operation in seawater, corresponding to –25 × demagnification of the ± 0.55 mm fiber tip deflection. A Strehl ratio of > 0.80 indicated diffraction-limited performance across the tissue-side FOV.

**Table 1:** Expected optical performance of miniaturized objective.

Expected optical performance	
Wavelength [nm]	1035
Numerical aperture	0.47
Demagnification	− 25
Field-of-view [μm^2^]	47 × 47
Working distance [μm]	112

The miniaturized objective contains two calcium fluoride (CaF_2_) aspheres, one Zinc sulfide (ZnS) asphere, a reflective microprism (Tower Optical Inc.) and a sapphire window (Tower Optical Inc.) (Fig. 1b). The three lenses image the scanned fiber tip onto a sub-epithelial tissue plane, the microprism folds the beam path 90° to enable radial focusing, and the sapphire window provides a tissue contact surface. As high pulse energies are required for sub-epithelial ablation, we needed to consider the nonlinear optical properties of select materials. Due to negligible multiphoton absorption in CaF_2_, we expect linear transmission of ≥ 12 μJ, 1 ps pulses, far higher than the pulse energies required for ablation^34^. This corresponds to a peak intensity of ~ 380 GW/cm^2^ at the fiber-side surface of the first CaF_2_ lens i.e., the smallest beam size at any surface within the objective. On the other hand, ZnS exhibits strong nonlinear refraction and absorption, however, the relatively low peak intensity (large beam waist) within the ZnS lens should mitigate these unwanted nonlinear effects. Specifically, the peak intensity of 0.26 GW/cm^2^ expected at the ZnS lens is lower than the experimentally determined threshold of 3.2 GW/cm^2^ required to initiate appreciable multiphoton absorption^29^.

Fabrication and assembly of the miniaturized objective was described previously by Jeon et al*.*^33^. The objective’s ~ 4.8 mm maximum clear aperture enabled the lenses to be packaged within a 6 mm diameter stainless-steel hypodermic tube (4TW, Vita Needle Company, Fig. 1c) and we used precision cut brass spacers to set appropriate distances between each lens.

### Mechanical design

**Figure 2:**
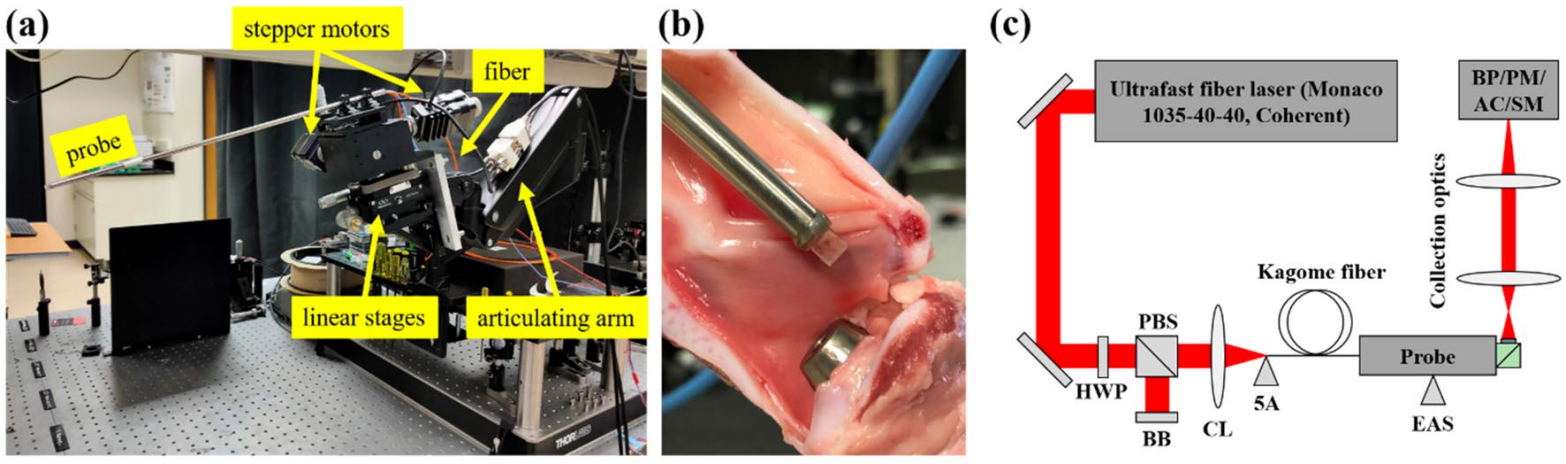
(**a**) Surgery system with probe integrated into external actuation system. (**b**) Probe tip placement on inferior porcine VF surface. (**c**) Experimental setup included a half-wave plate (HWP), polarizing beam splitter (PBS), beam block (BB), fiber coupling lens (CL), five-axis stage (5A), and external actuation system (EAS). Collection optics (CO) were modified based on the resolution and/or output beam size required for the beam profiler (BP), power meter (PM), autocorrelator (AC), or spectrometer (SM).

To enable large-area tissue ablation, we initially planned to mechanically scan the probe tip (i.e., the ablation FOV) using micromotors internally packaged within the probe. Specifically, a piezo-electric linear micromotor (SQL-RV-1.8, New Scale Technologies) and a brushless rotary micromotor (0602-B, MICROMO) packaged behind the piezo-fiber assembly would translate the probe tip, and, by extension, the ablation FOV, in a pseudo-raster pattern across a large sub-epithelial plane while maintaining a form factor suitable for laryngeal surgery. While this mechanical scanning system worked well during initial testing, it failed during ablation experiments. Due to its low stall torque, the linear micromotor was not able to move the probe tip while in contact with the tissue surface. In light of this problem, we opted to integrate the probe into an external actuation system (Fig. 2a). In this configuration, an articulating arm designed for a computer monitor was repurposed to enable flexible positioning of the surgery probe. The distal end of the arm holds two linear translation stages, a three-axis motorized stage (MAX302, Thorlabs), and the probe. The articulating arm and linear stages were used to position the probe tip on the vocal fold surface (Fig. 2b), while automated scanning of the ablation FOV across a large sub-epithelial plane was provided by translating the proximal end of the probe using two-stepper motors attached to the motorized stage. This configuration simplified the overall probe design, allowing the piezo-fiber and miniaturized objective subassemblies to be housed in a single ~ 30 cm long hypodermic tube. The overall length of the device is ~ 40 cm, similar to endoscopes currently used in laryngeal surgery, while the probe diameter of 6 mm allows for adequate vision clearance when operating within a rigid laryngoscope used for microlaryngeal surgery.

### Experimental setup

We utilized the experimental setup shown in Fig. 2c for probe characterization. We used a Yb fiber laser (40 W, 1035 ± 5 nm, 300 fs – 10 ps, 10 kHz – 50 MHz, Monaco 1035–40-40, Coherent Inc.) for all ablation experiments. Prior to experiments, we measured a laser beam quality factor of $M^{2} = 1.07 \pm 0.04$ using the method described by Siegman^35^. During ablation studies, we used either 1.1 ps or 5 ps pulses to avoid self-focusing when targeting sub-surface tissue structures within the highly scattering VF tissues^31^. Further, we used a laser repetition rate of 500 kHz to achieve high speed ablation while maintaining a low pulse overlap rate to avoid accumulative tissue heating effects^16,36–38^.

We used a bench-top nonlinear microscope to perform volumetric imaging of ablated VF tissues. Briefly, a Ti:sapphire laser oscillator (0.75 W, 775 nm, 100 fs, 80 MHz, Mai Tai, Spectra Physics) facilitates two-photon autofluorescence (TPAF) imaging across a 400 × 400 μm^2^ FOV when the laser beam is raster scanned onto the VF tissue by two galvanometric mirrors and expanded to the back aperture of a 20 × , 0.75 NA objective (Nikon Plan Apo, coverslip corrected). Emission is epi-collected, diverted through the appropriate filter (BG39, Schott), and detected by a photomultiplier tube (H7422-40, Hamamatsu). Images are generated by averaging 10 frames. The galvanometric mirrors, stage, and PMT are controlled and synchronized by MPScan software^39^.

## Results and discussion

### Kagome HCPCF characterization

**Figure 3:**
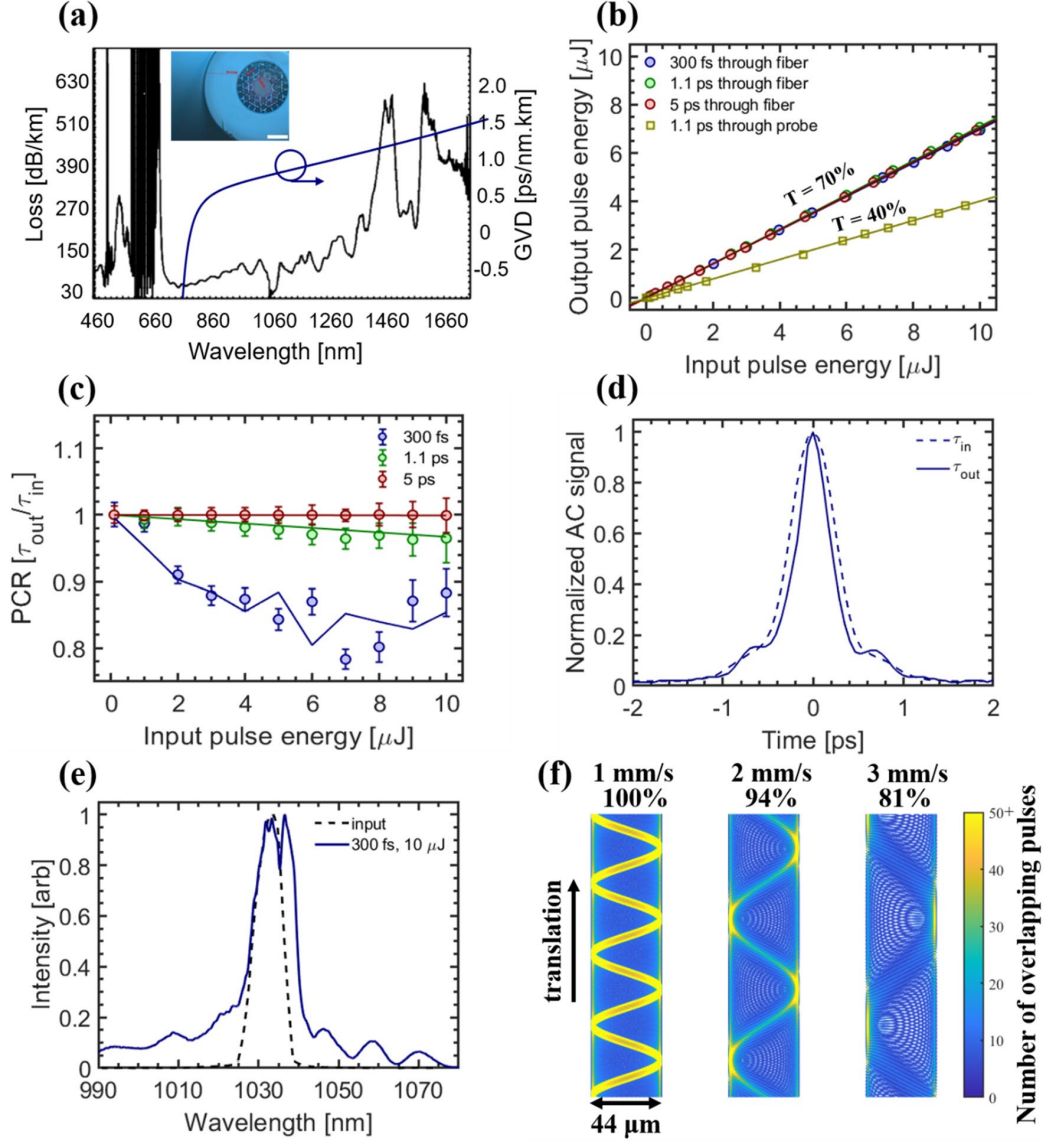
Kagome fiber characterization. (**a**) Measured loss and calculated disperion spectra of Kagome fiber. Inset shows *en face* view of fiber. Scale bar is 50 μm. (**b**) Measured transmission curves through 6 m long Kagome fiber for 300 fs, 1.1 ps, and 5 ps pulses and for the entire surgery probe at 1.1 ps. (**c**) Pulse compression ratio (PCR) at fiber output of 300 fs (blue), 1.1 ps (green), and 5 ps (red) pulses as a function of input pulse energy. Data points represent FHWM pulse widths measured with the autocorrelator (AC) while solid lines represent pulse widths predicted from simulations. Error bars represent 95% confidence intervals of sech^2^ fits to autocorrelator pulse width measurements. (**d**) Autocorrelator trace of 300 fs pulse at fiber input (dotted line) and output (solid line) for an input pulse energy of 10 μJ. (**e**) Pulse spectrum of 300 fs pulse at fiber input (dotted line) and output (solid line) for an input pulse energy of 10 μJ. (**f**) Lissajous scanning simulations showing the effect of lateral translation speed on pulse deposition profile. Translational speed of 1 mm/s results in complete coverage while 2 mm/s and 3 mm/s provide 94% and 81% coverage, respectively. The color bar to the right indicates the number of overlapping pulses throughout the scan area. Laser repetition rate was 500 kHz for data collected in (**b**–**e**).

We characterized the Kagome HCPCF using the experimental setup shown in Fig. 2c with the miniaturized objective removed. An *en face* view of the fiber and fiber loss/dispersion spectra are shown in Fig. 3a. The measured transmission was 70% when delivering 300 fs, 1.1 ps, and 5 ps pulses through the 6 m long fiber using a $f = 100$ mm coupling lens (Fig. 3b), representing ~ 81% coupling efficiency when considering fiber losses (0.1 dB/m at 1035 nm, Fig. 3a). We determined a fiber mode-field radius of $w_{MF} = 17 \pm 1$ μm by imaging the beam profile within the fiber core using a 40 × objective (0.75 NA, UPlanFLN, Olympus), beam profiler (SP928, Ophir Photonics), and an appropriate tube lens, agreeing well with the expected value of $w_{MF} = 18$ μm. We estimated the fiber’s numerical aperture by imaging the output beam profile at multiple axial locations in the far field, plotting the measured beam radii as a function of distance from the fiber tip, and calculating the corresponding divergence angle. The measured 0.022 ± 0.002 NA agreed favorably with the expected value of ~ 0.02.

To estimate the influence of chromatic dispersion and optical nonlinearities within the Kagome fiber, we characterized the pulse width and spectrum at the fiber input/output using a simulation and experimental-based approach. While linear dispersion and nonlinear effects should be minimal in the Kagome fiber, transmission of the high energy pulses required for surgery may still result in significant pulse distortion. Through the optical Kerr effect, a high-peak intensity pulse can modulate the instantaneous refractive index of the fiber, producing a nonlinear phase shift across the pulse. This effect, self-phase modulation (SPM), generates additional spectral components at the leading and trailing edges of the pulse as it travels along the fiber’s length and can accelerate pulse broadening in normally dispersive media. For an anomalously dispersive fiber, SPM can broaden, compress, or maintain the initial pulse width depending on the fiber geometry, length, and peak pulse intensity within the fiber.

We simulated pulse propagation through the Kagome fiber using the symmetric Fourier-split-step method (SFSS). Details on the SFSS method are described by Agrawal et al*.*^40^. In general, analytical solutions to Maxwell’s equations do not exist for nonlinear optical systems, and an approximate scalar form of the wave equation, the nonlinear Schröndinger equation, is commonly used to describe light propagation in waveguides,

1 $$ \frac{\partial A}{{\partial z}} + \frac{\alpha }{2}A + \frac{{i\beta_{2} }}{2}\frac{{\partial^{2} A}}{{\partial t^{2} }} - \frac{{\beta_{3} }}{6}\frac{{\partial^{3} A}}{{\partial t^{3} }} = i\gamma \left( {\left| A \right|^{2} A + \frac{i}{{\omega_{0} }}\frac{\partial }{\partial t}(\left| A \right|^{2} A)} \right) $$

where $\alpha$ is the linear absorption coefficient, $\beta_{2}$ and $\beta_{3}$ are second- and third-order dispersion coefficients, and $\omega_{0}$ is the angular frequency of the carrier wave. Equation (1) assumes the intensity of the pulse, $A(z,t)$, varies slowly with respect to wavelength (i.e., slowly varying envelope approximation). The nonlinear parameter, $\gamma$, is defined as $\gamma = {{\omega_{0} n_{2} } \mathord{\left/ {\vphantom {{\omega_{0} n_{2} } {cA_{eff} }}} \right. \kern-\nulldelimiterspace} {cA_{eff} }}$, where $c$ is the speed of light, $n_{2}$ is the nonlinear refractive index of air ($n_{2} = 3.01 \times 10^{ - 23} {\text{ m}}^{2} {\text{/W}}$ at 800 nm^41^), and $A_{eff}$ is the effective beam area defined by the fiber’s mode-field-radius. The first and second terms on the right hand side of Eq. (1) account for SPM and self-steepening, respectively. The field envelope of the input hyperbolic secant pulse is^40^:

2 $$ A(t) = \sqrt {P_{0} } {\text{sech}} (\frac{t}{{\tau_{0} }}) $$

where, $P_{0}$ is the pulse peak-power and $\tau_{0}$ is pulse width. The pulse width used in Eq. (2) can be converted to the full-width-at-half-maximum (FWHM) pulse width through the relation $\tau = 1.76\tau_{0}$, and $P_{0}$ is defined as^42^:

3 $$ P_{0} = 0.88\frac{E}{\tau } $$

where, *E* is the pulse energy. For our simulations, we assumed $\alpha = 0.023{\text{ m}}^{ - 1}$ and $\beta_{2} = - 4.37 \times 10^{ - 4} {\text{ ps}}^{2} {\text{/m}}$, as specified by the fiber manufacturer. We set $\beta_{3} = 0$, as third order dispersion is only significant near the zero dispersion wavelength, in our case ~ 730 nm, and because autocorrelator traces did not show the asymmetric trailing edge associated with third order dispersion^40^. We selected temporal and spatial step sizes based on methods outlined in^43^. High spatial and temporal discretization ensured simulation convergence and provided an accurate estimation of the output pulse shape.

We simulated pulse propagation through the Kagome fiber for three pulse widths: 300 fs, 1.1 ps, and 5 ps. Simulations predicted minimal pulse distortions when delivering 1.1 ps and 5 ps pulses through the Kagome fiber. For these longer pulse durations, peak intensities were low and nonlinear effects were negligible; temporal pulse profiles were practically unchanged up to an input pulse energy of 10 μJ (Fig. 3c). For 300 fs pulses, our model revealed changes in the output pulse width for increasing pulse energies, likely due to the interplay between SPM and anomalous dispersion in the Kagome fiber. Our simulations predicted a minimum pulse width of $\tau \sim 240{\text{ fs}}$ at 6 μJ and a recovery to $\tau \sim 260{\text{ fs}}$ at 10 μJ.

We then characterized the pulse width and spectra at the fiber input and output for a range of pulse energies using an autocorrelator (pulseCheck, APE GmbH) and a spectrometer (Mini4096CL, Avantes). For 1.1 ps and 5 ps pulses, we observed minimal variations in pulse width at the fiber output, agreeing favorably with simulations. For 300 fs pulses, we measured a minimum pulse width of $\tau \sim 235{\text{ fs}}$ at 7 μJ, followed by a recovery to $\tau \sim 265{\text{ fs}}$ at 10 μJ (Fig. 3c). In this case, output pulses developed “pedestals” at ≥ 9 μJ (Fig. 3d), leading to larger measurement uncertainties. Pedestals could have been caused by SPM-induced spectra which was uncompensated for in the anomalously dispersive Kagome fiber^44^. Indeed, we observed slight spectral broadening at the fiber output when using high energy 300 fs pulses, indicating the presence of SPM (Fig. 3e). In summary, the Kagome fiber provided efficient delivery of ultrashort pulses, and the minimal distortions observed during high pulse energy transmission should not significantly affect the surgical ablation capabilities of the probe.

With fiber characterization completed, we simulated the scanning performance of our surgical probe (Fig. 3f). Uneven weight distribution of the cantilevered fiber tip caused slight variations in the x- and y-axis resonance frequencies, naturally providing the requirements for Lissajous scanning. We utilized a Lissajous scanning algorithm to simulate the pulse deposition profile across the surgical ROI^27,29^. The Lissajous scan pattern at the tissue-side FOV is defined by:

4 $$ x(t) = \frac{1}{2}d_{x} \left[ {\sin (2\pi \nu_{x} t)} \right]; \, y(t) = \frac{1}{2}d_{y} [\cos (2\pi \nu_{y} t)] $$

where, $d_{x}$, $d_{y}$ are the x- and y-axis scan widths, and $\nu_{x}$, $\nu_{y}$ are x- and y-axis frequencies of the piezo-fiber scanning element, respectively. We simulated the scan pattern as the probe was translated in a single transerve direction to estimate the pulse deposition profile while advancing the surgical probe at a constant speed. For the expected surgery parameters: ablation FOV 47 × 47 µm^2^, focal beam radius $w_{0} = 0.94$ μm, $v_{x} ,v_{y} = 942{\text{ Hz, 895 Hz}}$, laser repetition rate of 500 kHz, and ≤ 2 mm/s translation speed, we estimated at least 94% of the FOV will receive at least one pulse, with a median value of at least 15 overlapping pulses per spot.

### Miniaturized objective testing

We characterized the miniaturized objective using the setup shown in Fig. 2c. Transmission efficiency through the entire probe was 40%, slightly lower than the expected 46% (Fig. 3b). Importantly, transmission through the objective remains linear for high pulse energies, indicating negligible multiphoton absorption within the lenses. Initial spot size measurements were performed by imaging the objective’s focal plane when delivering a collimated beam from our fiber laser towards the objective’s back aperture, similar to studies described by Jeon et al*.*^33^. Briefly, a Keplerian telescope reduced the input beam radius to ~ 250 μm, and a five-axis stage was used to align the objective along the beam path. In this case, Zemax simulations predicted a 1/*e*^2^ focal spot radius, $w_{0}$, of 0.95 μm for focusing in seawater. We measured $w_{0}$ by imaging the miniaturized objective’s focal plane onto the beam profiler using a 20 × water immersion objective (0.95 NA, XLUMPlanFl, Olympus) and a 400 mm tube lens. To coalign the focal plane of the miniaturized objective with the object plane of the 20 × objective, we first imaged the surface of a 170 μm thick borosilicate glass coverslip using the imaging setup and then translated the miniaturized objective towards the coverslip until the smallest focal spot was observed on the beam profiler. A water droplet was placed between the probe tip and coverslip to mimic focusing in tissue. The ~ 42 × magnification of this setup, determined by imaging 10 μm features on a micrometer slide (#53–713, Edmund Optics), in addition to the beam profiler’s small pixel size (3.69 μm), provided an accurate estimation of the focal spot size. We determined $w_{0} = 1.15 \pm 0.09$ μm, corresponding to a Strehl ratio of ~ 0.81 and indicating diffraction-limited performance.

**Figure 4:**
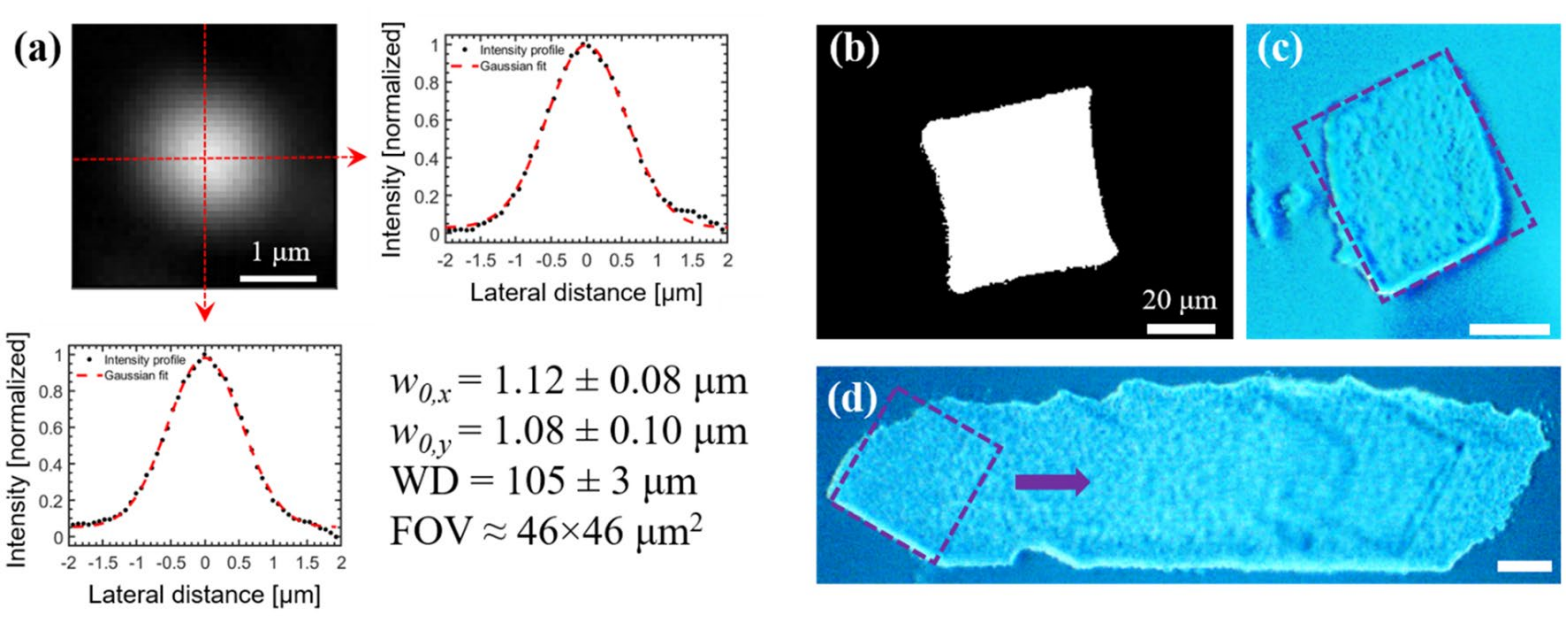
Optical performance of surgical probe. (**a**) Focal spot of miniaturized objective imaged with 20 × water immersion objective, 400 mm tube lens, and beam profiler. Beam profile was fit to Gaussian function to determine 1/*e*^2^ focal beam radius ($w_{0}$). Uncertainties represent 95% confidence intervals for the Gaussian fit to the vertical and horizontal profiles. Scale bar is 1 μm. (**b**) Binary image of 46 × 46 μm^2^ FOV at the probe focal plane. (**c**) Ablation of borosilicate glass coverslip with surgical probe. The size of the ablation FOV created on the coverslip agrees well with FOV imaging results. (**d**) Scanning the coverslip laterally enables formation of an ablation trench across the glass surface. Scale bars are 20 μm.

We performed similar experiments with this imaging setup after integrating the miniaturized objective with the piezo-fiber and mechanical scanning assemblies. Initially, we determined the ideal distance between the fiber tip and first CaF_2_ lens by carefully adjusting the fiber position until the smallest focal spot was observed on the beam profiler. We found a fiber-lens distance of 2 – 3 mm provided the best performance, agreeing well with the 2 mm distance specified by the Zemax prescription. When using a custom nylon spacer to set the fiber-lens distance to 2 mm in the final probe assembly, we determined $w_{0} = 1.12 \pm 0.10$ μm (Fig. 4a), approximately 19% larger than the Zemax estimate of 0.94 μm. Discrepancies in focal plane spot size may be attributed to slight ellipticity in the focused beam (i.e., comatic and/or astigmatic aberrations caused by tilt of the fiber with respect to the objective’s optical axis). We determined a working distance of ~ 105 μm by recording the translation distance required to produce a sharp image of the probe tip relative to its focal plane using a manual linear stage attached to the probe, agreeing well with the 112 μm working distance predicted by Zemax. Scanning the fiber tip ± 0.53 mm produced an ablation FOV of ~ 46 × 46 μm^2^ (± 0.4 μm), corresponding to an approximately –23 × demagnification, which agreed well with the expected system demagnification of –25 × (Fig. 4b).

We performed preliminary glass ablation studies to assess the surgical capabilities of the probe. We coaligned the probe’s focal plane to the top surface of a glass coverslip using a modified focal plane imaging setup, consisting of the 40 × air immersion objective, a 50 mm tube lens, and a CCD camera (Manta G-201, Allied Vision). A water droplet was placed between the probe tip and coverslip to mimic focusing in tissue, similar to focal spot measurements described above. Careful alignment was necessary to initiate ablation as bubble formation, caused by beam focusing in water, restricted laser power delivery to the coverslip surface. When carefully focused on the coverslip surface, an ablation FOV of ~ 45 × 48 μm^2^ (Fig. 4c) could be obtained using 360 nJ pulses, corresponding to a fluence that is ~ 3.5 × greater than the single pulse ablation threshold of borosilicate glass^45^. A mechanical shutter controlled the laser exposure time, equal to 0.5 s for single FOV ablation. Finally, we ablated a trench on the coverslip surface by holding the coverslip stationary and laterally translating the probe tip across a 2 mm distance at 1 mm/s (Fig. 4d) while opening the shutter for a short time during the middle of the scan.

### Tissue ablation and biomaterial injection studies

We characterized the tissue ablation properties of the probe using excised porcine larynges. To select appropriate laser operating parameters for surgery, namely the pulse energy at the epithelial surface required to initiate ablation within the SLP layer of the VF, a priori knowledge of the VF optical scattering properties is required. Thus, we measured scattering lengths and ablation thresholds of porcine VF using an ultrafast laser ablation-based technique^46^. Fresh porcine larynges were purchased from a local slaughterhouse and tested within 4 h of animal sacrifice. For scattering length and ablation threshold measurements, we extracted rectangular sections of the inferior VF mucosa from the mid-VF by dissecting the mucosa from the underlying muscle. As previous studies indicated that both tissue dehydration and storage in saline may significantly influence optical scattering^23,47,48^, we decided to test fresh VF samples without saline immersion immediately after excision to better mimic in vivo conditions.

The surface pulse energy, $E_{surf}$, required to initiate optical breakdown and form ablation voids within the SLP can be described by^46^:

5 $$ E_{surf} = F_{th,SLP} \pi w_{0}^{2} e^{{\left[ {\frac{{z_{ep} }}{{\ell_{s,ep} }} + \frac{{z_{0} }}{{\ell_{s,SLP} }}} \right]}} $$

Here, $F_{th,SLP}$ is the SLP fluence threshold, $\ell_{s,ep}$ and $\ell_{s,SLP}$ are the epithelial and SLP scattering lengths, $z_{ep}$ is the epithelial thickness, and $z_{0} = z - z_{ep}$ is the distance within the SLP. Following the procedure described in^46^, we determined $\ell_{s,ep} = 147.2 \pm 15.7$ μm, $\ell_{s,SLP} = 53.8 \pm 3.4$ μm, $F_{th,ep} = 1.55 \pm 0.08$ J/cm^2^, and $F_{th,SLP} = 1.44 \pm 0.15$ (n = 3). Assuming a target depth $z = 105$ μm and an average porcine VF epithelial thickness of ~ 60 μm^23,46,48^, we estimated $E_{surf} \sim 200$ nJ is required to exceed $F_{th,SLP}$. We used methods reported by Subramanian et al*.* to estimate the effects of self-focusing during sub-surface ablation in porcine VF^30^. We determined that focal plane fluences must be lower than $F = 25F_{th,SLP}$ to ensure peak powers are lower than the critical power for self-focusing, assuming a 0.47 NA and 105 μm working distance of the miniaturized objective as well as a 1.1 ps pulse width of the fiber laser. To ensure ablation and avoid self-focusing, we targeted focal plane fluences $F = 10F_{th,SLP}$, thus $E_{surf} \sim 2$ μJ at the probe tip or pulse energies of ~ 5 μJ at the Kagome fiber input when considering transmission losses through the fiber and objective.

We assessed the probe’s ablation performance on excised porcine hemilarynges. We created sub-epithelial voids within the SLP by scanning the probe tip across the medial VF surface using the external actuation system described in Sect. “Mechanical design”. Specifically, we moved the probe tip in the x-direction in increments of $h_{x}$ after every ± 2 mm translation in the y-direction to scan the ablation FOV in a pseudo-raster pattern across a large sub-epithelial plane.

During initial ablation experiments we noticed a defect within the optical adhesive (NOA-61, Norland Products) used to secure the sapphire window to the prism. While the fluence of ≤ 2 mJ/cm^2^ at this interface was well below the expected single pulse femtosecond laser damage threshold of the adhesive (that can be approximated by the ablation threshold of polymers, 1 – 2.6 J/cm)^49^, we suspect impurities within the adhesive act as localized hot spots that can lead to accumulation of structural defects when exposed to the high peak powers/high peak intensities required for tissue ablation. To test this issue further, we prepared NOA-61 samples and exposed them to peak laser intensities which were similar to those expected in the miniaturized objective. Indeed, we found that defects appeared within the NOA-61 samples after 5 min exposure when using 1.1 ps, 400 nJ pulses focused to a spot radius of 35 μm (10 mJ/cm^2^, 2 × 10^10^ W/cm^2^). Reducing the pulse width from 1.1 ps to 500 fs (i.e., increasing the peak intensity while keeping fluence constant) resulted in damage in < 2 min, suggesting that onset of damage is indeed related to peak intensity and exposure time. On the other hand, we determined that 5 ps pulses caused no damage within the NOA-61 samples after 30 min, even when using 3 × higher fluences. Thus, to avoid further damage to the objective, we used 5 ps pulses for all subsequent tissue ablation experiments. To remove the defect within the miniaturized objective, we separated the sapphire window from the prism, cleaned the left over NOA-61 residue from both mating surfaces, and re-adhered the window to the prism surface. Encouragingly, transmission, spot size, working distance, and FOV measurements were similar to values obtained prior to damage, suggesting that the objective can perform as designed.

**Figure 5:**
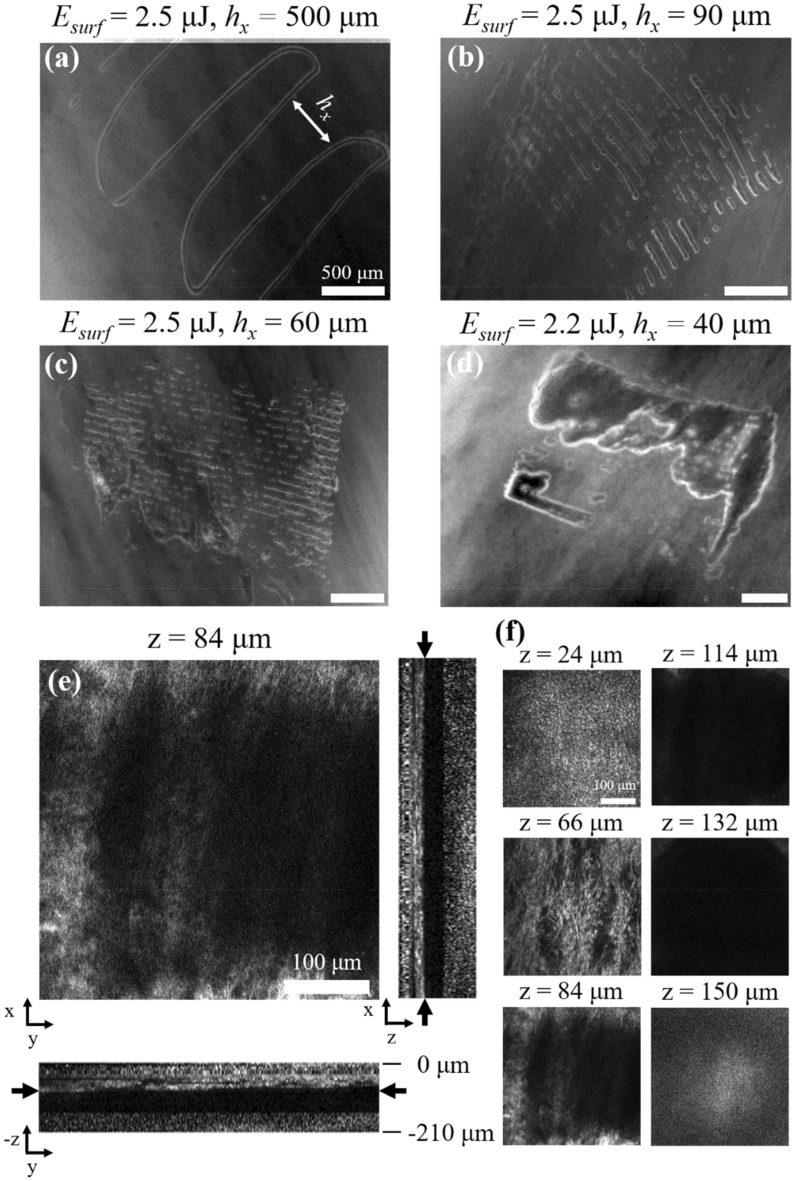
Tissue ablation performance of surgical probe. Sub-epithelial ablation with (**a**) $E_{surf} = 2.5$ μJ, $h_{x} = 500$ μm, (**b**) $E_{surf} = 2.5$ μJ, $h_{x} = 90$ μm, (**c**) $E_{surf} = 2.5$ μJ, $h_{x} = 60$ μm, and (**d**) $E_{surf} = 2.2$ μJ, $h_{x} = 40$ μm. Scale bars are 500 μm. (**e**) TPAF images of sub-epithelial void created with $E_{surf} = 2.2$ μJ, $h_{x} = 40$ μm. Center image shows the top of the void 84 μm deep into VF, well within the SLP. Cross sections through the centerlines of the center image are shown below and to the right. Black arrows in cross section images indicate the imaging plane shown in center image. (**f**) Montage of selected frames from the z-stack. Epithelial cells are visible up to a depth of 60 μm and collagen fibers are visible at deeper depths. The void is centered at 114 μm and extends ± 30 μm. Scale bars are 100 μm.

To determine the optimal laser fluence and x-axis step size, $h_{x}$, required for large area sub-epithelial ablation, we performed several experiments with different parameters. We found that these parameters must be carefully selected to avoid ablation of the tissue surface. For example, surface ablation was almost always observed when using $E_{surf} \sim 3.6$ μJ, corresponding to focal plane fluences of $\sim 18F_{th,SLP}$, regardless of $h_{x}$. When $E_{surf} = 2 - 2.5$ μJ (i.e., $F = 10F_{th,SLP} - 13F_{th,SLP}$) and $h_{x}$ is large, each y-axis scan created a narrow sub-surface ablation “line” with a width approximately equal to the ablation FOV width (Fig. 5a – c). We found that $E_{surf} = 2.2$ μJ, $h_{x} = 40$ μm provided the most uniform coverage of the targeted region without causing damage to the tissue surface (Fig. 5d). In this case, $h_{x}$ was smaller than the ablation FOV width and ablation lines overlapped to produce a large, continuous void. As shown in Fig. 5e and f, voids were localized well below the epithelium, providing a space for therapeutic biomaterial injection. In this case, the void was centered ~ 114 μm below the tissue surface, agreeing well with the measured working distance of ~ 105 μm.

**Figure 6:**
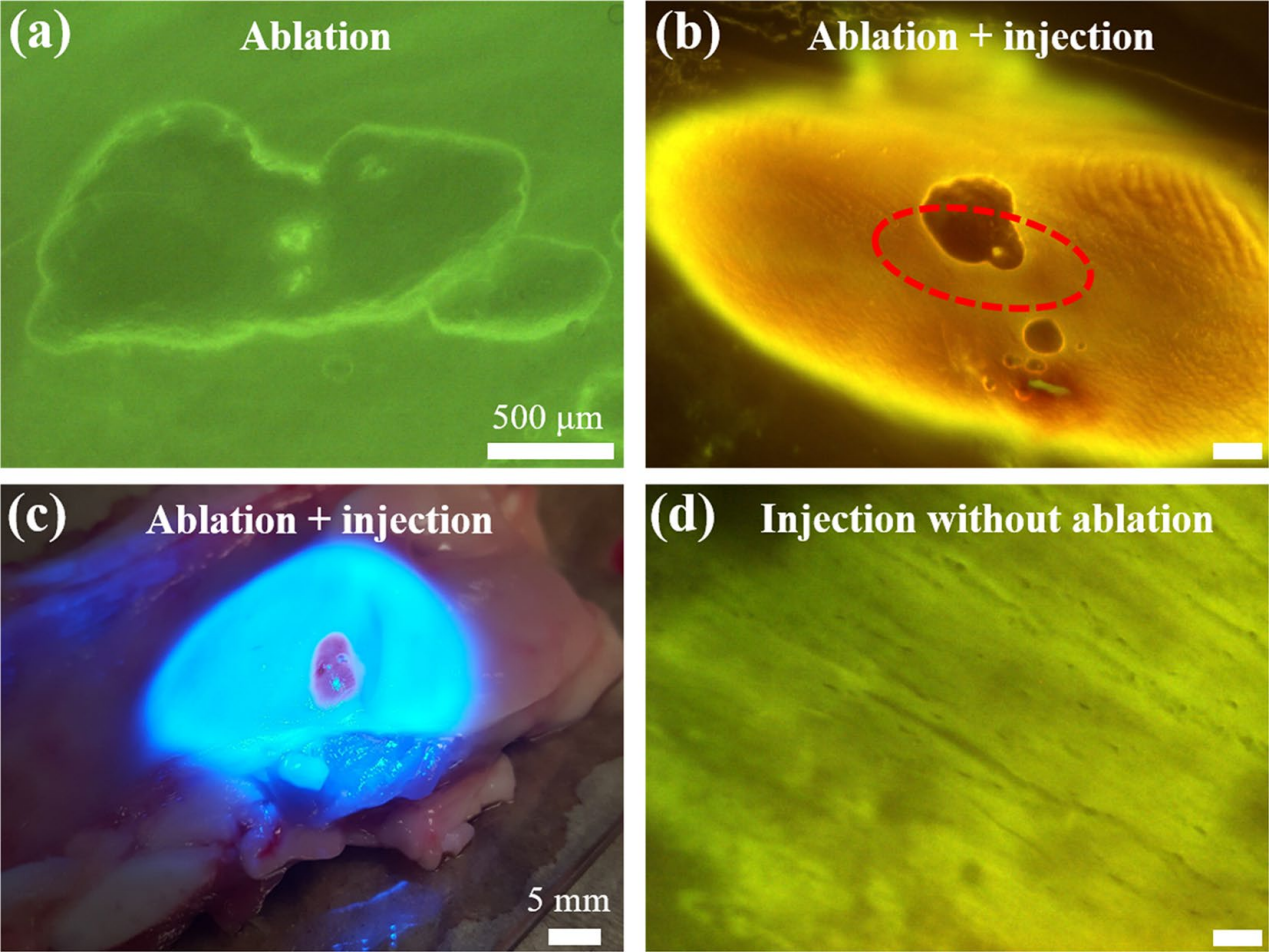
Biomaterial injection into a sub-epithelial void created with surgery probe in excised porcine VF. (**a**) Image of a ~ 1 × 2 mm^2^ sub-epithelial void created using surface pulse energies of $E_{surf} \sim 2.2$ μJ and x-axis step sizes of $h_{x} = 40$ μm. Total surgery time was ~ 3 min. (**b**) Void shown in (**a**) after biomaterial injection. The tissue surface was rinsed with saline and wiped with lens tissue prior to imaging. The injected biomaterial can be seen as the brightly fluorescent volume that remains localized underneath the tissue surface in the sub-epithelial ablation region, shown in (**a**). The red dotted oval indicates the void size before injection. The ablation void increases in size as the biomaterial is injected into the void. (**c**) Image of the entire porcine hemilarynx taken with camera phone after biomaterial injection shown in (**b**). The red region is the injected biomaterial localized within the void. The bright blue region is light from the stereo-microscope. (**d**) Fluorescence image of biomaterial injection into VF without ablation. Most of the injected volume backflows onto tissue surface. After rinsing and wiping of the tissue surface, we see that a small amount of the biomaterial has non-selectively diffused into the surrounding tissues. Scale bars are 500 μm in (**a**), (**b**), (**d**) and 5 mm in (**c**).

We injected a Rhodamine-tagged polyethylene glycol (PEG30) hydrogel into treated (i.e., ablated) and untreated porcine VF to demonstrate improved biomaterial localization within sub-epithelial voids created with the probe. Large voids were created using the aforementioned setup and $E_{surf} \sim 2.2$ μJ, $h_{x} = 40$ μm (Fig. 6a). After ablation, hemilarynges were transferred to a custom injection setup, consisting of a pressure regulator and a solenoid valve attached to a 0.5 mL syringe containing the biomaterial. To precisely control the injection volume, a custom LabView program controlled the flow duration by opening/closing the solenoid valve to momentarily pressurize the syringe and dispense a small volume (~ 10 μL) through a 23 G hypodermic needle near the void. We used a three-axis micromanipulator (KITE-L, World Precision Instruments LLC) to insert the needle tip beneath the epithelium near the ablated void, and a fluorescent stereo-microscope for visualization during the injection process. After injection, the tissue surface was rinsed with saline and wiped with lens tissue. The biomaterial was successfully localized within the sub-epithelial void (Fig. 6b and 6c). The injected volume expands into the sub-epithelial space with negligible backflow onto the VF surface. In contrast, biomaterial injected into untreated VF (i.e., without ablation) tends to backflow and deposit on the VF surface. After rinsing and wiping, we see that a small amount of the injected volume is non-selectively diffused into VF tissue surrounding the injection site (Fig. 6d).

## Conclusions

We presented a miniaturized ultrafast laser surgery system designed to treat VF scarring. A Kagome HCPCF allowed for flexible delivery of high energy ultrashort laser pulses to a custom-built 6 mm diameter objective, producing a 1.12 ± 0.10 μm focal beam radius across a ~ 46 × 46 μm^2^ ablation FOV. The tight beam focusing provided by the probe, in addition to a ~ 105 μm working distance, enabled sub-surface focusing and ablation beneath the VF epithelium. Towards a clinically viable surgery system, we integrated the probe into an external actuation system, consisting of an articulating mechanical arm and multiple high precision automated stages, which should enable flexible positioning of the probe during future in vivo studies.

We assessed the probe’s surgical performance by performing parametric ablation studies on fresh porcine hemilarynges. We found that laser parameters must be carefully selected during surgery to avoid damage to the tissue surface and the probe itself. Further, automated scanning of the probe tip/ablation FOV across the tissue surface/sub-epithelial plane enabled creation of large, continuous voids. Specifically, we could create large voids when using 5 ps, 2.2 μJ pulses and translating the ablation FOV in 40 μm increments across a 1 × 2 mm^2^ area. We confirmed that ablation voids were localized below the epithelium; TPAF imaging indicated that voids were centered ~ 114 μm below the tissue surface, agreeing well with the Zemax design and working distance measurements. Finally, we demonstrated localization of a model biomaterial within sub-epithelial voids created with the miniaturized probe, suggesting that the surgical system presented here may be used to treat vocal fold scarring in a clinical setting. Future studies will integrate the probe into a clinically ready workstation to assess the viability of our vocal fold scarring therapy in vivo.

## Data Availability

Data supporting the findings of this study are available from the corresponding author on request.
